# Supplementary material for: ER Unfolded Protein Response in Liver In Vivo Is Characterized by Reduced, Not Increased, De Novo Lipogenesis and Cholesterol Synthesis Rates with Uptake of Fatty Acids from Adipose Tissue: Integrated Gene Expression, Translation Rates and Metabolic Fluxes
Source: Int J Mol Sci. 2022 Jan 19;23(3):1073. doi: 10.3390/ijms23031073 (PMC8835023; doi:10.3390/ijms23031073)
Supplement: Supplementary file 1 [file ijms-23-01073-s001.zip › ijms-1541806-supplementary.pdf]

Supplementary Figures

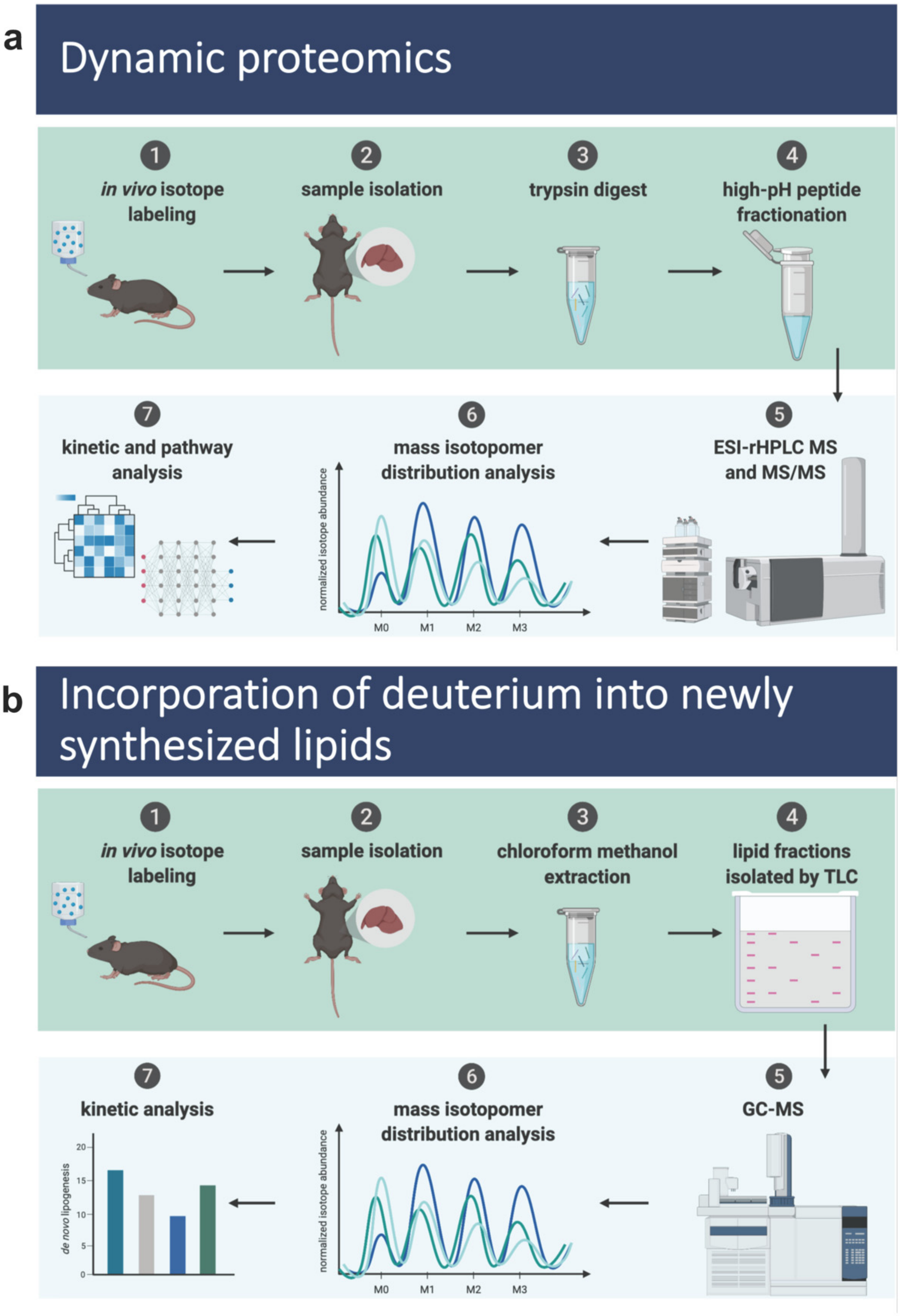

Figure S1. (a) Experimental overview of dynamic proteomics approach. (b) Experimental overview of measurement of *de novo* lipogenesis.

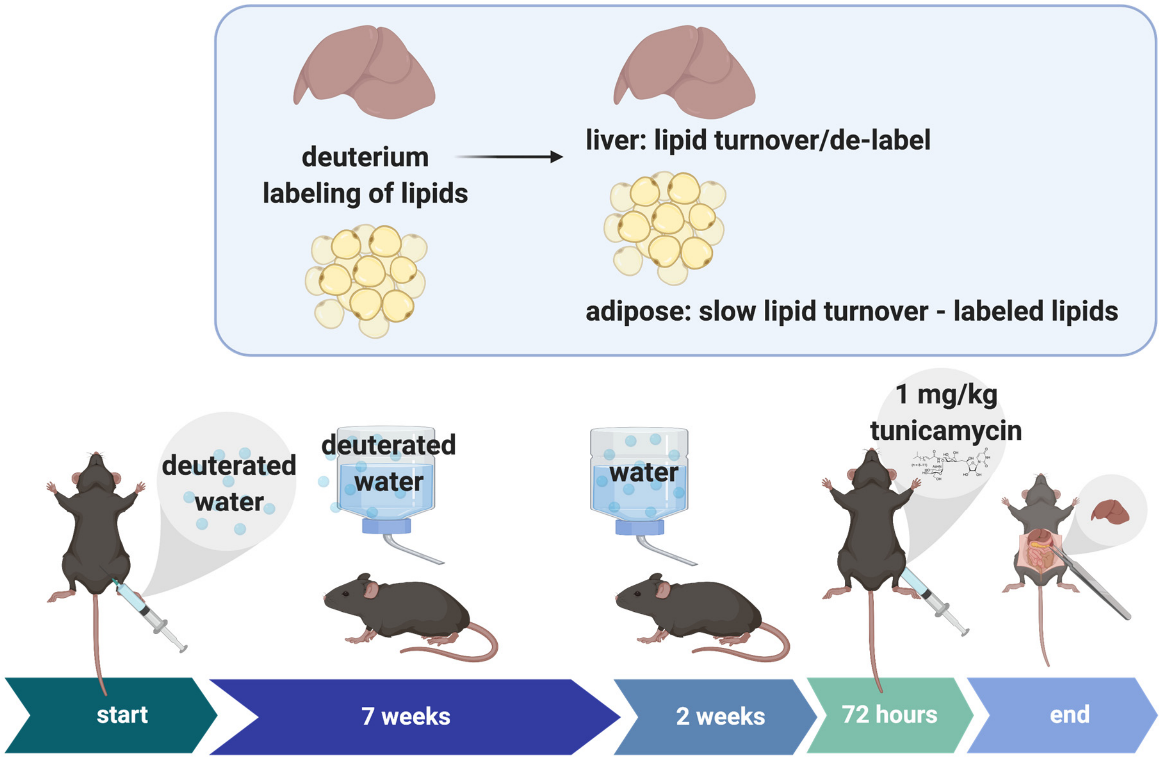

Figure S2. Experimental overview of pre-labeling experiment.

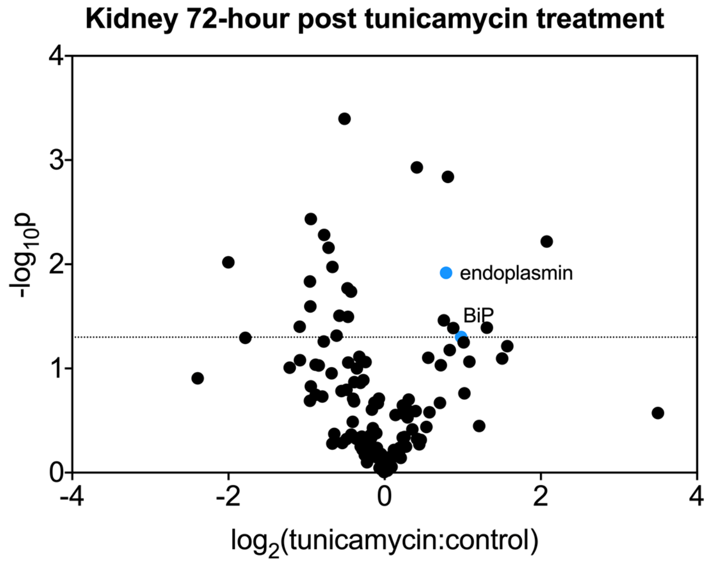

Figure S3. Ratio of tunicamycin:control treated mice kidneys. Volcano plot of all proteins for which fractional synthesis rates were measured 72 hrs post tunicamycin treatment. Points expressed as log2 fold-change tunicamycin treated/control on x-axis and - log10(p-value), obtained from 2-tail t-test, on y-axis.

Table S1. Individual protein synthesis rate ratios of tunicamycin/control mice. Ratio of above 1 indicates a higher synthesis rate under ER stress. Ratio of below 1 indicates a lower synthesis rate under ER stress.

Protein	6 hr ratio	12 hr ratio	24 hr ratio	48 hr ratio	72 hr ratio
10 kDa heat shock protein, mitochondrial	0.63	0.79	1.41	1.03	1.10
14-3-3 protein beta/alpha	1.00	0.55	1.09	0.96	1.01
14-3-3 protein epsilon	1.47	0.27	0.21	0.63	0.76
14-3-3 protein gamma	3.82	0.56	1.05	1.01	1.08
14-3-3 protein zeta/delta	1.25	0.81	1.32	1.16	1.29
2-aminomuconic semialdehyde dehydrogenase	0.91	1.02	0.32	0.79	0.92
2-hydroxyacyl-CoA lyase 1	1.16	0.81	1.12	1.11	0.97
2-iminobutanoate/2-iminopropanoate deaminase	0.72	0.58	0.58	0.59	0.88
2,4-dienoyl-CoA reductase, mitochondrial	1.20	0.38	0.36	0.95	1.03
3-hydroxyanthranilate 3,4-dioxygenase	0.62	0.56	0.33	0.75	0.55
3-hydroxyisobutyrate dehydrogenase, mitochondrial	1.00	0.80	1.08	1.30	1.08
3-ketoacyl-CoA thiolase A, peroxisomal	0.53	0.52	0.77	0.86	0.98
3-ketoacyl-CoA thiolase B, peroxisomal	0.61	0.34	0.69	0.68	0.94
3-ketoacyl-CoA thiolase, mitochondrial	0.81	1.55	0.69	0.57	0.81
4-aminobutyrate aminotransferase, mitochondrial	1.62	0.54	1.51	0.66	
4-hydroxyphenylpyruvate dioxygenase	0.58	0.72	0.84	0.89	0.87

4-trimethylaminobutyraldehyde dehydrogenase	0.42	0.52	1.26	0.79	0.83
40S ribosomal protein S17	0.73	0.73	0.63	1.48	0.68
40S ribosomal protein S2			0.66	1.48	1.11
40S ribosomal protein S20		0.35	0.59	1.47	
40S ribosomal protein S28	0.35	1.08	1.46	1.25	1.63
40S ribosomal protein S3			1.10		
40S ribosomal protein S3a	1.23	1.29	0.87	1.10	1.25
40S ribosomal protein S4, X isoform			3.49		
40S ribosomal protein S7	0.51	0.96	1.18	1.27	1.24
40S ribosomal protein S8	0.73	4.60	1.09	1.15	1.29
40S ribosomal protein SA			1.18	1.06	1.52
6-phosphogluconate dehydrogenase, decarboxylating	0.96	1.56	0.94	0.82	0.88
6-phosphogluconolactonase	0.54	0.34	1.11	0.88	1.01
60 kDa heat shock protein, mitochondrial	0.60	1.36	0.95	1.00	1.24
60S acidic ribosomal protein P0	0.31			2.01	
60S ribosomal protein L12		0.24	1.38	2.00	1.54
60S ribosomal protein L23a	0.70			1.19	
60S ribosomal protein L27a			0.07	0.28	
60S ribosomal protein L4			3.62		
60S ribosomal protein L5	1.21		0.30		
60S ribosomal protein L6	0.72	0.82	1.09	1.29	1.30
Acetyl-CoA acetyltransferase, cytosolic	1.19	0.27	0.51	0.62	0.78
Acetyl-CoA acetyltransferase, mitochondrial	0.43	0.43	0.91	0.52	1.47

Aconitate hydratase, mitochondrial	1.12	1.21	1.62	0.86	0.79
Actin, alpha cardiac muscle 1	1.51	3.96	0.98	1.07	1.04
Actin, aortic smooth muscle	0.92	0.91	0.59	0.92	1.17
Actin, cytoplasmic 1	0.69	2.99	0.92	0.99	1.04
Acyl-CoA synthetase family member 2, mitochondrial	1.37	2.07	1.31	0.66	1.10
Acyl-CoA-binding protein	0.75	0.41	0.54	0.59	0.73
Acyl-coenzyme A synthetase ACSM1, mitochondrial	0.72	1.38	0.70	1.01	0.82
Adenosine kinase	0.67	0.74	0.91	1.00	1.21
Adenosylhomocysteinase	0.53	1.92	0.94	0.98	1.06
Adenylate kinase 2, mitochondrial	0.95	0.61	0.82	0.74	0.65
ADP/ATP translocase 2	0.98	0.93	0.76	0.76	0.93
Alanine aminotransferase 1	0.60	0.95	1.21	1.03	0.84
Alcohol dehydrogenase 1	0.61	0.68	0.49	0.33	0.52
Alcohol dehydrogenase class-3	0.98	0.94	1.02	0.65	0.90
Aldehyde dehydrogenase family 3 member A2	0.74	0.55	0.89	0.89	0.79
Aldehyde dehydrogenase, cytosolic 1	0.74	1.37	0.62	0.67	0.62
Aldehyde dehydrogenase, mitochondrial	0.54	0.79	0.80	0.72	0.86
Aldehyde oxidase 3	0.92	0.93	0.55	0.72	0.61
Aldo-keto reductase family 1 member A1	1.33	1.19	1.31	0.75	0.98
Aldo-keto reductase family 1 member C13	0.83	6.33	0.59	0.53	
Alpha-aminoadipic semialdehyde dehydrogenase	0.87	0.64	0.65	0.63	0.69

Alpha-aminoadipic semialdehyde synthase, mitochondrial	0.80	0.91	1.15	0.93	1.19
Alpha-enolase	0.65	1.09	0.59	0.67	0.81
Annexin A5	0.92			1.17	
Arginase-1	0.55	1.44	0.53	0.88	1.02
Argininosuccinate lyase	1.08	1.93	0.57	0.60	0.79
Argininosuccinate synthase	0.56	0.60	0.89	1.18	1.12
Aspartate aminotransferase, cytoplasmic	1.03	2.95	1.64	1.70	1.72
Aspartate aminotransferase, mitochondrial	0.74	1.94	0.93	0.85	1.07
ATP synthase F(0) complex subunit B1, mitochondrial			0.37	0.47	0.95
ATP synthase subunit alpha, mitochondrial	0.72	0.59	0.84	0.81	0.89
ATP synthase subunit beta, mitochondrial	0.59	2.33	0.53	0.78	1.01
ATP synthase subunit d, mitochondrial	0.63	0.45	0.76	0.78	0.90
ATP synthase subunit delta, mitochondrial	0.87	1.00	0.60	1.44	0.89
ATP synthase subunit O, mitochondrial	2.12	1.02	0.61	0.71	1.01
ATP-citrate synthase	0.83			0.54	
Beta-actin-like protein 2	0.80	2.73	0.91	0.92	1.03
Betaine--homocysteine S-methyltransferase 1	0.60	1.47	0.71	1.24	0.94
Bifunctional epoxide hydrolase 2	0.70	0.80	1.15	0.89	0.98
Bile acyl-CoA synthetetase	5.48	0.24	0.40	0.96	1.00

C-1-tetrahydrofolate synthase, cytoplasmic	1.24	1.24	0.57	0.49	1.11
Calcium-binding mitochondrial carrier protein Aralar2	1.25	2.21	0.88	0.66	1.04
Calreticulin	1.45	2.36	2.38	1.96	1.63
Carbamoyl-phosphate synthase [ammonia], mitochondrial	0.63	1.08	0.57	0.66	0.74
Carbonic anhydrase 3	0.76	0.68	0.29	0.24	0.36
Carboxylesterase 1D	0.48	0.58	0.29	0.26	0.40
Carboxylesterase 1F	0.66	0.32	0.39	0.36	0.43
Carboxylesterase 3A	0.58	0.59	0.62	0.45	0.56
Carboxylesterase 3B	0.63	0.66	0.63	0.54	0.54
Catalase	0.59	1.23	0.65	0.86	0.94
Catechol O-methyltransferase	0.70	1.31	0.49	0.80	
Clathrin heavy chain 1	1.49	0.65	2.30	1.53	1.11
Cystathionine gamma-lyase	0.87	0.94	1.06	1.43	1.28
Cysteine sulfinic acid decarboxylase	0.75	0.66	0.37	0.35	0.46
Cytochrome b-c1 complex subunit 1, mitochondrial	0.95	0.70	0.97	0.70	0.87
Cytochrome b-c1 complex subunit 2, mitochondrial			0.44		
Cytochrome b-c1 complex subunit 6, mitochondrial	0.44	0.65	0.54	0.71	0.77
Cytochrome b5	0.79	1.59	0.91	0.95	1.10
Cytochrome c oxidase subunit 2	0.55	1.31	0.95	0.84	1.04
Cytochrome c oxidase subunit 4 isoform 1, mitochondrial	0.80			0.96	

Cytochrome c1, heme protein, mitochondrial	0.65	1.90		1.39	0.86
Cytochrome P450 1A2	0.50	0.69	0.95		
Cytochrome P450 2A12	1.01		1.43		1.23
Cytochrome P450 2C29	0.51	0.57	0.47	0.42	0.73
Cytochrome P450 2D10	0.65	0.74	0.89	0.79	1.24
Cytochrome P450 2D26	0.75	0.53	1.21	1.04	1.24
Cytochrome P450 2D9	0.75	0.85	0.94	0.76	1.21
Cytochrome P450 2E1	0.73	0.91	0.97	1.04	0.99
Cytochrome P450 2F2	0.57	0.59	0.69	0.51	0.76
Cytochrome P450 3A11	0.70	0.80	0.83	0.92	1.02
Cytoplasmic aconitate hydratase	0.78	0.72	0.46	0.57	1.00
Cytosol aminopeptidase	1.04	0.71	0.87	0.95	1.23
Cytosolic 10-formyltetrahydrofolate dehydrogenase	0.45	1.11	0.55	0.74	0.89
D-beta-hydroxybutyrate dehydrogenase, mitochondrial	0.61	0.43	0.67	0.60	0.95
D-dopachrome decarboxylase	0.81	1.72	1.11	1.17	1.00
Delta-1-pyrroline-5-carboxylate dehydrogenase, mitochondrial	0.94	2.14	0.68	0.71	0.91
Dihydrolipoyl dehydrogenase, mitochondrial	1.28	0.65	1.42	0.61	1.22
Dihydropteridine reductase	1.73	0.48	0.79	0.82	
Dihydropyrimidinase	0.50	0.52	0.47	0.40	
Dimethylaniline monooxygenase [N-oxide-forming] 5	0.77	0.83	0.73	0.72	0.83
Dimethylglycine dehydrogenase, mitochondrial	2.95	0.69	0.74	0.78	0.72

Electron transfer flavoprotein subunit alpha, mitochondrial	1.16	0.80	0.74	0.75	0.82
Electron transfer flavoprotein subunit beta	0.79	0.86	1.03	0.70	0.85
Electron transfer flavoprotein-ubiquinone oxidoreductase, mitochondrial				0.82	
Elongation factor 1-alpha 1	0.55	0.68	0.90	0.88	1.26
Elongation factor 1-alpha 2	0.75		1.24	0.85	1.73
Elongation factor 1-beta	1.09	1.00	1.99	0.94	
Elongation factor 1-delta	0.96	1.25	1.16	1.54	1.42
Elongation factor 1-gamma	4.50	0.87	1.23	1.46	1.33
Elongation factor 2	0.53	0.60	0.89	0.84	1.08
Endoplasmic reticulum chaperone BiP	4.00	3.10	2.70	1.93	1.75
Endoplasmin	2.73	3.13	2.89	1.86	1.60
Enoyl-CoA delta isomerase 1, mitochondrial	0.26	0.95	1.21	0.84	0.92
Enoyl-CoA hydratase, mitochondrial	1.85		2.30	0.25	0.72
Epoxide hydrolase 1	0.81	0.70	0.69	0.69	0.80
Estradiol 17 beta-dehydrogenase 5	0.44	1.68	0.49	0.30	0.54
Farnesyl pyrophosphate synthase	0.80	1.50	1.18	1.49	1.12
Fatty acid synthase	0.52	0.57	0.42	0.37	0.71
Fatty acid-binding protein, liver	0.58	0.89	0.28	0.33	0.52
Ferritin light chain 1	0.63	1.10	1.15	1.13	1.04
Fibrinogen gamma chain	1.09		2.26	2.53	1.93
Flavin reductase (NADPH)	1.39	0.14	0.98	0.50	0.96

Formimidoyltransferase-cyclodeaminase	0.80	1.27	1.05	0.98	1.03
Fructose-1,6-bisphosphatase 1	0.81	0.67	1.11	1.45	1.32
Fructose-bisphosphate aldolase B	0.69	0.84	1.05	1.08	1.02
Fumarate hydratase, mitochondrial	0.58	1.00	1.20	0.87	1.30
Fumarylacetoacetase	0.75	0.70	0.75	0.88	1.00
Glucose-6-phosphate isomerase			1.39	0.45	0.68
Glutamate dehydrogenase 1, mitochondrial	0.57	0.84	0.44	0.43	0.56
Glutamine synthetase	0.82	1.33	0.64	0.37	0.28
Glutaryl-CoA dehydrogenase, mitochondrial	0.98	0.50	0.95	1.69	1.09
Glutathione peroxidase 1	0.92	0.61	0.44	0.61	0.62
Glutathione S-transferase A1				0.25	0.68
Glutathione S-transferase A3	0.46	1.40	0.51	0.45	0.59
Glutathione S-transferase A4	0.50	0.52	0.62	0.29	0.44
Glutathione S-transferase Mu 1	0.69	3.79	1.23	0.96	0.95
Glutathione S-transferase Mu 2	0.13	0.58	1.35	0.97	0.99
Glutathione S-transferase Mu 3	0.51	3.66	1.31	0.99	1.06
Glutathione S-transferase P 1	0.74	2.34	0.72	0.40	0.49
Glutathione S-transferase P 2	0.68	1.85	0.61	0.38	0.59
Glyceraldehyde-3-phosphate dehydrogenase	0.54	0.59	1.19	0.87	1.11
Glycerol-3-phosphate dehydrogenase [NAD(	0.26				
Glycine N-acyltransferase	1.73	1.81	1.11	0.76	0.97
Glycine N-methyltransferase	0.66	1.33	0.94	1.15	1.23

Glycogen phosphorylase, liver form	0.87	0.60	0.70	0.42	0.62
Glyoxylate reductase/hydroxypyruvate reductase	0.58	0.50	0.73	0.94	0.95
GTP:AMP phosphotransferase AK3, mitochondrial			0.24	0.23	1.82
Heat shock 70 kDa protein 1-like		0.90	1.25	0.88	1.74
Heat shock cognate 71 kDa protein	0.93	0.58	0.92	1.07	1.01
Heat shock protein HSP 90-alpha	0.73	0.31	0.72	0.81	0.95
Heat shock protein HSP 90-beta	0.70	0.61	0.77	0.97	0.93
Hemoglobin subunit alpha	1.36	1.37	1.14	1.41	0.44
Hemoglobin subunit beta-1	0.78	4.18	1.36	0.47	0.53
Hemoglobin subunit beta-2	0.36	1.25	1.73	0.21	0.42
Histidine ammonia-lyase	0.61	0.71	0.52	0.67	0.93
Histidine triad nucleotide-binding protein 1	0.92	1.08	1.17	1.20	1.26
Homogentisate 1,2-dioxygenase	0.84	0.60	0.50	0.69	0.82
Hydroxyacyl-coenzyme A dehydrogenase, mitochondrial	1.33	0.85	0.46	0.62	0.56
Hydroxymethylglutaryl-CoA lyase, mitochondrial	0.63	2.54	1.01	0.78	1.12
Hydroxymethylglutaryl-CoA synthase, mitochondrial	0.60	2.19	0.80	0.86	1.12
Hypoxia up-regulated protein 1	0.51	1.49	2.93	2.04	1.73
Indolethylamine N-methyltransferase	1.99	0.62	0.38	0.30	0.41
Interleukin-12 receptor subunit beta-2	0.97	0.81	0.74	0.57	0.90
Isochorismatase domain-containing protein 2A	0.52	1.27	1.09	1.64	1.38

Isocitrate dehydrogenase [NADP] cytoplasmic	1.62	0.91	0.77	0.65	0.90
Isovaleryl-CoA dehydrogenase, mitochondrial	0.42		0.56	1.00	0.95
L-lactate dehydrogenase A chain	0.81	0.40	0.44	0.74	1.40
Lactoylglutathione lyase	0.44	0.57	0.55	0.47	0.80
Lipoamide acyltransferase component of branched-chain alpha-keto acid dehydrogenase complex, mitochondrial		0.36	0.82	1.09	1.52
Liver carboxylesterase 1	0.32	0.86	0.58	0.26	0.44
Lon protease homolog, mitochondrial				4.83	
Long-chain-fatty-acid--CoA ligase 1	0.77	0.84	0.35	0.48	0.67
Lysine-specific demethylase 5A	0.92				
Macrophage migration inhibitory factor	0.25	0.30	1.56	1.23	1.22
Major urinary protein 1	0.81	0.96	0.79	0.91	0.86
Major urinary protein 11	0.79	0.96	0.97	0.87	0.99
Major urinary protein 17	0.80	0.95	1.01	0.94	0.84
Major urinary protein 18	0.82	0.96	0.76	0.97	0.83
Major urinary protein 2	0.84	0.95	0.79	0.92	0.93
Malate dehydrogenase, cytoplasmic	0.99	1.99	1.14	1.27	1.10
Malate dehydrogenase, mitochondrial	0.85	0.45	1.57	1.43	1.38
Maleylacetoacetate isomerase	0.60	0.91	0.89	0.99	1.00
Mediator of RNA polymerase II transcription subunit 23			0.42	0.21	
Medium-chain specific acyl-CoA dehydrogenase, mitochondrial	0.48	0.61	0.94	0.87	1.02

Membrane-associated progesterone receptor component 1	0.79	0.37	1.17	0.53	1.22
Methanethiol oxidase	0.73	0.62	0.37	0.40	0.44
Methylmalonate-semialdehyde dehydrogenase [acylating], mitochondrial	1.17	0.89	0.86	0.94	1.20
Methyltransferase-like protein 7B			2.30	1.52	1.32
Microsomal glutathione S-transferase 1	1.07	0.60	0.53	0.42	
Microsomal triglyceride transfer protein large subunit	2.54		0.95		
Microtubule-associated protein 2		0.90	1.03	1.35	1.25
Multiple epidermal growth factor-like domains protein 8	1.38	0.74	0.96	0.45	0.73
NAD-dependent protein deacetylase sirtuin-3		1.28	0.44	0.59	
NADH dehydrogenase [ubiquinone] 1 alpha subcomplex subunit 9, mitochondrial			0.79	0.87	
NADH-cytochrome b5 reductase 3	1.03	0.09	0.51	0.50	0.75
NADH-ubiquinone oxidoreductase 75 kDa subunit, mitochondrial	0.85			0.89	
NADPH--cytochrome P450 reductase	0.88	1.20	0.68	0.64	
Nesprin-1				0.45	
Nicotinate-nucleotide pyrophosphorylase [carboxylating]	0.85	0.25	0.73		0.89
Non-specific lipid-transfer protein	0.59	0.56	0.60	0.57	0.81
Nucleoside diphosphate kinase A	0.73			1.23	
Nucleoside diphosphate kinase B	0.74	0.56	1.86	1.43	1.34

Ornithine aminotransferase, mitochondrial	0.59	0.67	0.87	0.94	1.06
Ornithine carbamoyltransferase, mitochondrial	1.16	2.37	0.36	0.35	0.53
Peptidyl-prolyl cis-trans isomerase A	0.83	0.39	1.02	0.77	0.95
Peptidyl-prolyl cis-trans isomerase B			1.45	1.55	1.22
Peroxiredoxin-1	0.69	0.51	0.59	0.76	1.01
Peroxiredoxin-2	0.46	0.17	0.78	0.68	1.33
Peroxiredoxin-5, mitochondrial	1.12	1.98	0.73	0.73	0.81
Peroxiredoxin-6	0.86	0.78	0.72	0.67	0.81
Peroxisomal acyl-coenzyme A oxidase 1	0.62	0.71	0.86	1.13	1.29
Peroxisomal bifunctional enzyme	1.06	0.80	0.66	1.10	1.13
Peroxisomal multifunctional enzyme type 2	1.10	0.51	1.16	1.35	1.59
Phenazine biosynthesis-like domain-containing protein 1	0.69	0.84	0.77	0.62	0.63
Phenazine biosynthesis-like domain-containing protein 2	0.70	0.81	0.62	0.71	0.69
Phenylalanine-4-hydroxylase	0.56	0.72	0.99	0.88	1.09
Phosphoglucomutase-1	0.80	0.47	0.81	0.82	1.06
Phosphoglycerate kinase 1	0.81	0.86	1.27	1.18	1.26
Phosphoglycerate mutase 1	0.84	0.49	1.27	0.72	0.91
Polyubiquitin-B	0.74	1.07	1.06	1.10	1.22
Pregnancy zone protein	0.63			1.23	
Probable helicase senataxin	0.44	0.44		0.38	0.63
Probable imidazolonepropionase				1.21	
Profilin-1	0.65	1.65			

Prohibitin	0.55	0.54	1.80	0.93	0.84
Protein ABHD14B		0.70	0.82		0.85
Protein disulfide-isomerase	0.92	1.36	2.10	1.68	1.52
Protein disulfide-isomerase A3	1.18	2.20	2.33	1.62	1.51
Protein disulfide-isomerase A4	2.26	1.88	2.14	2.11	1.89
Protein disulfide-isomerase A6	1.49	1.68	1.92	2.21	1.55
Protein NDRG2	0.94			0.92	
Protein PRRC2A					
Protein/nucleic acid deglycase DJ-1	1.92		1.10		0.95
Pyrethroid hydrolase Ces2a	0.57	0.59	0.63	0.42	0.76
Pyruvate carboxylase, mitochondrial	0.72	0.79	0.83	0.82	1.07
Pyruvate kinase PKLR	0.54	2.33	1.09	0.40	
Rab GDP dissociation inhibitor beta	1.27	0.93	1.06		
Radixin				1.07	
Ras-related protein Rab-1A	0.94			1.09	
Regucalcin	0.88	2.28	0.61	0.33	0.46
Retinal dehydrogenase 1	0.53	1.46	0.56	0.67	0.77
Rho GDP-dissociation inhibitor 1	0.47	2.28	0.78	0.75	0.90
S-adenosylmethionine synthase isoform type-1	0.65	0.71	1.34	1.18	1.21
S-formylglutathione hydrolase	0.97	0.71	0.99	1.02	1.14
Sarcosine dehydrogenase, mitochondrial	0.81	0.81	0.92	1.02	1.17
SEC14-like protein 2	0.84	0.77	1.04	0.74	0.90
Selenium-binding protein 2	0.61	0.79	0.41	0.39	0.41
Sepiapterin reductase			1.35	1.00	1.28

Serine protease inhibitor A3K	0.34	0.37	0.46	0.50	0.38
Serine protease inhibitor A3M	0.32	0.33	0.25	0.45	0.05
Serotransferrin	0.35	0.28	0.45		0.85
Serum albumin	0.34	1.87	0.51	0.39	0.67
Short-chain specific acyl-CoA dehydrogenase, mitochondrial	1.00	0.68	1.27	0.67	0.70
Sorbitol dehydrogenase	0.71	0.68	0.60	0.65	0.85
Staphylococcal nuclease domain-containing protein 1	0.47	0.21	1.92	2.10	1.22
START domain-containing protein 10	1.32	0.29	0.66	0.77	
Stress-70 protein, mitochondrial	0.84	1.13	1.92	1.22	1.21
Succinate dehydrogenase [ubiquinone] flavoprotein subunit, mitochondrial	0.74	0.55	0.68	0.88	0.93
Succinate--CoA ligase [GDP-forming] subunit beta, mitochondrial	1.12	0.78	0.97	0.94	0.93
Succinate-semialdehyde dehydrogenase, mitochondrial	1.29	0.53	0.38	0.60	0.88
Superoxide dismutase [Cu-Zn]	0.80	2.34	0.52	0.71	0.87
Superoxide dismutase [Mn], mitochondrial	1.60		0.98	0.58	0.81
T-complex protein 1 subunit gamma	1.02	2.47	1.00	0.97	0.80
T-complex protein 1 subunit zeta	0.92	0.54	1.02	0.99	0.96
Thioredoxin domain-containing protein 12	0.45	0.32	0.35	0.53	0.58
Thiosulfate sulfurtransferase	0.64	0.85	0.82	0.74	0.85
Trans-1,2-dihydrobenzene-1,2-diol dehydrogenase		0.66	0.78	0.69	1.04

Transitional endoplasmic reticulum ATPase	1.08	1.95	1.59	0.95	1.29
Transketolase	0.89	1.34	0.68	0.60	0.55
Trifunctional enzyme subunit alpha, mitochondrial	0.97	2.78	0.66	0.85	0.96
Trifunctional enzyme subunit beta, mitochondrial	0.62	0.90	0.73	0.92	0.96
Triokinase/FMN cyclase	0.75	0.98	0.70	0.65	0.82
Triosephosphate isomerase	0.76	0.83	0.60	0.78	0.78
Tripeptidyl-peptidase 1	5.36			0.14	
Tubulin alpha-1A chain		0.96	1.17	0.80	0.86
Tubulin alpha-1B chain	1.09	0.84	1.09	0.73	0.66
Tubulin alpha-1C chain	1.03	0.72	0.92	0.69	0.71
Tubulin alpha-4A chain	0.75	1.48	0.93	0.69	0.61
Tubulin alpha-8 chain	0.60	1.07	0.65		0.61
Tubulin beta-2A chain	0.85	1.30	1.38	1.23	1.09
Tubulin beta-2B chain					1.36
Tubulin beta-4A chain		1.28	2.31	1.19	1.22
Tubulin beta-4B chain	1.11	1.45	1.49	1.38	1.42
Tubulin beta-5 chain	2.20	1.48	1.63	1.18	1.68
Ubiquitin-40S ribosomal protein S27a	0.53	0.63	1.25	0.49	1.10
Ubiquitin-like modifier-activating enzyme 1	0.78	1.16	1.01	1.11	1.22
UDP-glucose 6-dehydrogenase	1.52	0.72	0.48	0.54	0.87
UDP-glucuronosyltransferase 1-1	0.69	0.82	0.78	1.14	1.30
UDP-glucuronosyltransferase 1-6		0.30	0.92		1.37
UDP-glucuronosyltransferase 1-9	0.55	1.21	1.15	1.10	1.29

UDP-glucuronosyltransferase 2B17	0.58	0.80	0.63	0.94	1.02
Uricase	0.59	0.60	0.88	0.96	
Urocanate hydratase	0.59	0.58	0.37	0.52	0.40
UTP--glucose-1-phosphate uridylyltransferase	0.74	0.91	1.22	1.05	1.07
Valacyclovir hydrolase	0.67	0.60	0.48	0.56	0.72
Very long-chain acyl-CoA synthetase	0.99	0.71	0.61	0.90	0.99
Very long-chain specific acyl-CoA dehydrogenase, mitochondrial	0.86	1.21	0.84	0.87	1.08
Vitamin D-binding protein	0.68	0.86	0.92	4.66	
